# P-1374. Immunological Evaluation of Individuals with Multidrug-Resistant Tuberculosis in Callao, Peru

**DOI:** 10.1093/ofid/ofaf695.1561

**Published:** 2026-01-11

**Authors:** David M Henson, Yiyi Luo, Natalia Egri, Milagros Zavaleta, Daniel Cuadras, Manel Juan, Rocio Egoavil-Espejo, Daniel Rojas, Walter Portugal, Paolo Miotto, Daniella Bartholomeu, Jorge Sanchez, Stefan Niemann, Jorge Alarcon, Laia Alsina, Ana Esteve-Sole, Moises A Huaman

**Affiliations:** University of Kentucky, Lexington, KY; Institut de Recerca Sant Joan de Déu, Barcelona (IRSJD), Barcelona, Catalonia, Spain; Hospital Clinic, Barcelone, Catalonia, Spain; Centro de Investigaciones Tecnológicas, Biomedicas y Medioambientales, Callao, Peru, Callao, Callao, Peru; IQVIA, Barcelona, Catalonia, Spain; SJD Barcelona Children’s Hospital and Hospital Clínic, Barcelona, Catalonia, Spain; University of Cincinnati, Cincinnati, Ohio; Centro de Investigaciones Tecnológicas, Biomedicas y Medioambientales, Callao, Callao, Peru; Direccion Regional de Salud del Callao, Callao, Callao, Peru; IRCCS San Raffaele Scientific Institute, Milan, Italy, Milan, Lombardia, Italy; Universidade Federal Minas Gerais, Brazil, Belo Horizonte, Minas Gerais, Brazil; Centro de Investigaciones Tecnológicas, Biomedicas y Medioambientales, Callao, Peru, Callao, Callao, Peru; Molecular and Experimental Mycobacteriology, Research Center Borstel; German Center for Infection Research, Partner Site Hamburg-Lübeck-Borstel-Riems, Borstel, Germany, Borstel, Schleswig-Holstein, Germany; Universidad Mayor de San Marcos, Lima, Lima, Peru; Allergy and Clinical Immunology Department, Hospital Sant Joan de Déu Institut de Recerca Sant Joan de Déu, University of Barcelona, Barcelona, Spain, Barcelona, Catalonia, Spain; National Institutes of Health, Bethesda, Maryland; Division of Infectious Diseases, University of Cincinnati, Cincinnati, USA, Cincinnati, OH

## Abstract

**Background:**

*Mycobacterium tuberculosis* (*Mtb*) causes significant morbidity and mortality with worse outcomes in individuals infected with multi-drug resistant *Mtb* (MDR-TB). Specific MDR-TB strains have shown additional virulence factors that could prompt altered host immune response in comparison to drug susceptible *Mtb* (DS-TB). We evaluated the immune response of individuals with MDR-TB in Callao, Peru compared to DS-TB.

**Figure:**
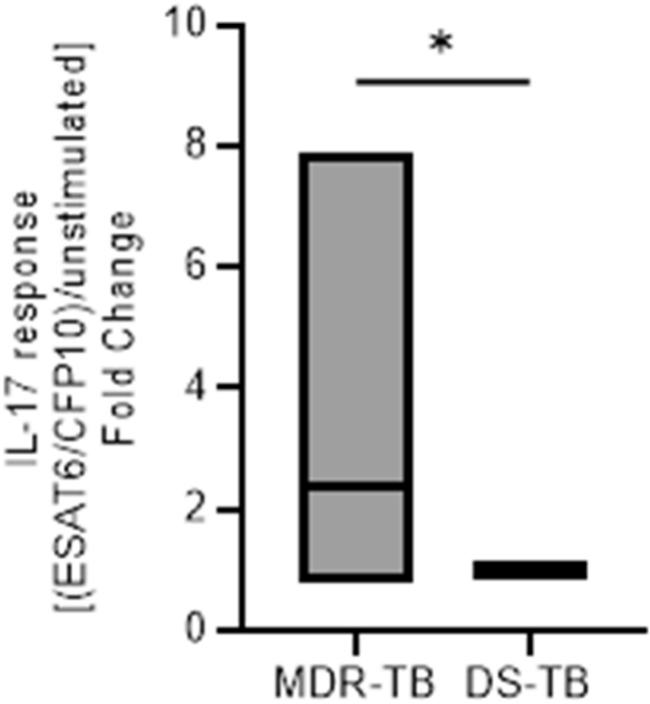
Mtb-specific IL-17 production from PBMC collected from participants newly diagnosed with MDR-TB and DS-TB. Boxes show mean, min, and max values of fold change in IL-17 between ESAT-6·CFP-10-stimulated vs. unstimulated conditions. *p < 0.05

**Methods:**

Adults with a new diagnosis of MDR-TB and DS-TB were enrolled from tuberculosis clinics in Callao, Peru while healthy control participants were recruited from local medicine clinics and community. Participants provided blood samples for peripheral blood mononuclear cells (PBMCs) and plasma isolation. Soluble immune markers including proinflammatory cytokines and immunoglobulins were tested in plasma. PBMCs were analyzed via flow cytometry for immunophenotyping and via cell stimulation assays. To account for false positive rates with 75 distinct cell populations in the immunophenotyping assay Benjamini-Hochberg correction was employed.

**Results:**

Analysis of cell phenotypes identified notable differences. CD8+ T cells were increased in patients with MDR-TB compared to DS-TB (medians (interquartile range) [n]: 51.9 (31.2-65.7) [14] and 27.8 (23.2-34.8) [22], p = 0.036)), and the frequency of CD8+/CD38+ cells were decreased in MDR-TB compared to DS-TB (4.47 (3.3-6.6) [14] and 9.4 (6.2-13.9) [22], p = 0.0057). While analysis of soluble plasma factors did not show significant differences, analysis of cell stimulation assays showed an increased IL-17 production in PBMC from participants with MDR-TB compared to DS-TB when stimulated with the *Mtb* antigen ESAT6/CFP10 (see Figure; p=0.015).

**Conclusion:**

While much of the immune response to MDR-TB appears similar to DS-TB, we found notable differences, with increased CD8+ T cells, decreased CD8/CD38+ T cells, and increased IL-17 production in response to *Mtb* stimulation. These data suggest that MDR-TB could prompt differential immune signatures, including expanded IL-17 responses. Further work may lead to better understanding of host-pathogen interactions in MDR-TB disease and possible optimized therapies.

**Disclosures:**

All Authors: No reported disclosures

